# Inverse Perfusion Requirements of Supra- and Infratentorial Brain Metastases Formation

**DOI:** 10.3389/fneur.2018.00391

**Published:** 2018-05-30

**Authors:** Tanja Schneider, André Kemmling, Julian Schroeder, Klaus Pantel, Markus Glatzel, Gerhard Schoen, Malte Mohme, Jens Fiehler, Susanne Gellißen

**Affiliations:** ^1^Department of Diagnostic and Interventional Neuroradiology, University Medical Center Hamburg-Eppendorf, Hamburg, Germany; ^2^Department of Radiology, Schoen Klinik Hamburg Eilbek, Hamburg, Germany; ^3^Department of Neuroradiology, University Medical Center Schleswig-Holstein, Luebeck, Germany; ^4^Department of Neurology, University Medical Center Hamburg-Eppendorf, Hamburg, Germany; ^5^Center for Experimental Medicine, Institute of Tumor Biology, University Medical Center Hamburg-Eppendorf, Hamburg, Germany; ^6^Center for Diagnostics, Institute of Neuropathology, University Medical Center Hamburg-Eppendorf, Hamburg, Germany; ^7^Department of Medical Biometry and Epidemiology, University Medical Center Hamburg-Eppendorf, Hamburg, Germany; ^8^Department of Neurosurgery, University Medical Center Hamburg-Eppendorf, Hamburg, Germany

**Keywords:** blood-brain barrier, cerebral blood flows, hemodynamics, MR tomography, multidetector computed tomography, neoplasm metastases, neoplasms, perfusion imaging

## Abstract

**Background and Aims:** Vascular border zones and the gray-white matter junction are preferred sites for the development of brain metastases (BM), whereas microvascular lesions are known to be a protective factor. In this proof of concept study, we aim to study the relationship of blood perfusion and the spatial distribution of BM.

**Materials and Methods:** An average CT perfusion atlas of 107 healthy patients was created. Voxel-wise reference perfusion values were extracted from BM-negative and BM-positive regions in a second cohort of 100 untreated patients harboring 809 BM confirmed by MRI. A comparison of regional perfusion values was performed using the independent *t*-test.

**Results:** In contrast to supratentorial BM that develop preferably in areas with lower CBV/CBF and longer MTT/TTP compared to the average regional perfusion (*p* < 0.001), infratentorial BM showed a higher CBV/CBF and shorter MTT/TTP (*p* < 0.001).

**Conclusion:** Our results imply differing pathophysiological mechanisms underlying supra- and infratentorial BM spreading. The inverse perfusion patterns may result from differences in vascular supply, hemodynamic requirements, and/or production of pro-angiogenic factors.

## Introduction

In principal, there are two main hypotheses on the development of brain metastases (BM). The “seed and soil” hypothesis states that metastatic cells favor a particular biochemical environment or tend to colonize selectively in suitable tissues (soils) due to cell surface and endothelium properties (1, 2). Furthermore, the “anatomical-mechanical” theory proposes that dissemination of metastases follows arterial vessel caliber and local blood flow (3).

Prior studies already demonstrated that the presence and the amount of small vessel ischemic disease are protective factors against the development of BM (4–7). Hwang et al. found a higher propensity of BM in vascular border zones and the gray-white matter junction (8). These findings suggest a relevant impact of brain perfusion on BM formation.

Here, we aim to study the relationship of the spatial distribution of the BM of 100 untreated patients and the underlying blood perfusion pattern. We hypothesize that all BM would preferably develop in areas where physiological perfusion is rather high compared to areas without any BM. We further hypothesize that central necrotic BM tend to colonize in areas with less perfusion.

## Materials and methods

This single center, retrospective study was conducted in compliance with the local ethics committee (Ethik-Kommission der Aerztekammer Hamburg, WF-018/15) with a waiver of informed consent.

### CT perfusion atlas

Cerebral blood flow in cancer patients has been shown to be altered due to paraneoplastic changes, chemotherapy administration, neoangiogenesis, and/or disruption of the blood-brain barrier (9, 10). Therefore, we used pooled whole-brain CT perfusion datasets of a reference population. The perfusion atlas was generated from 107 patients who were triaged by CT perfusion for symptoms of transient ischemic attack but without evidence of infarction or any perfusion abnormality, symptoms on follow up, or vascular abnormality that was verified by MRI 24 h later (11). Quantitative perfusion maps were obtained for cerebral blood volume (CBV in ml/100 g brain tissue), cerebral blood flow (CBF in ml/100 g brain tissue/min), mean transit time (MTT in s), and time to peak (TTP in s). CT perfusion was performed on a Somatom Flash scanner (Siemens Healthcare, Erlangen, Germany) by applying the following scan parameters: 80 kV, 200 mAs, collimation 2 × 64 × 0.6 mm, 5 mm slice reconstruction, 1.5 s sampling rate for 45 s, biphasic i.v. injection with 30 ml of 400 mM/ml iodinated contrast medium followed by 30 ml NaCl chaser bolus with a flow rate of 4 ml/s.

**Figure 1:**
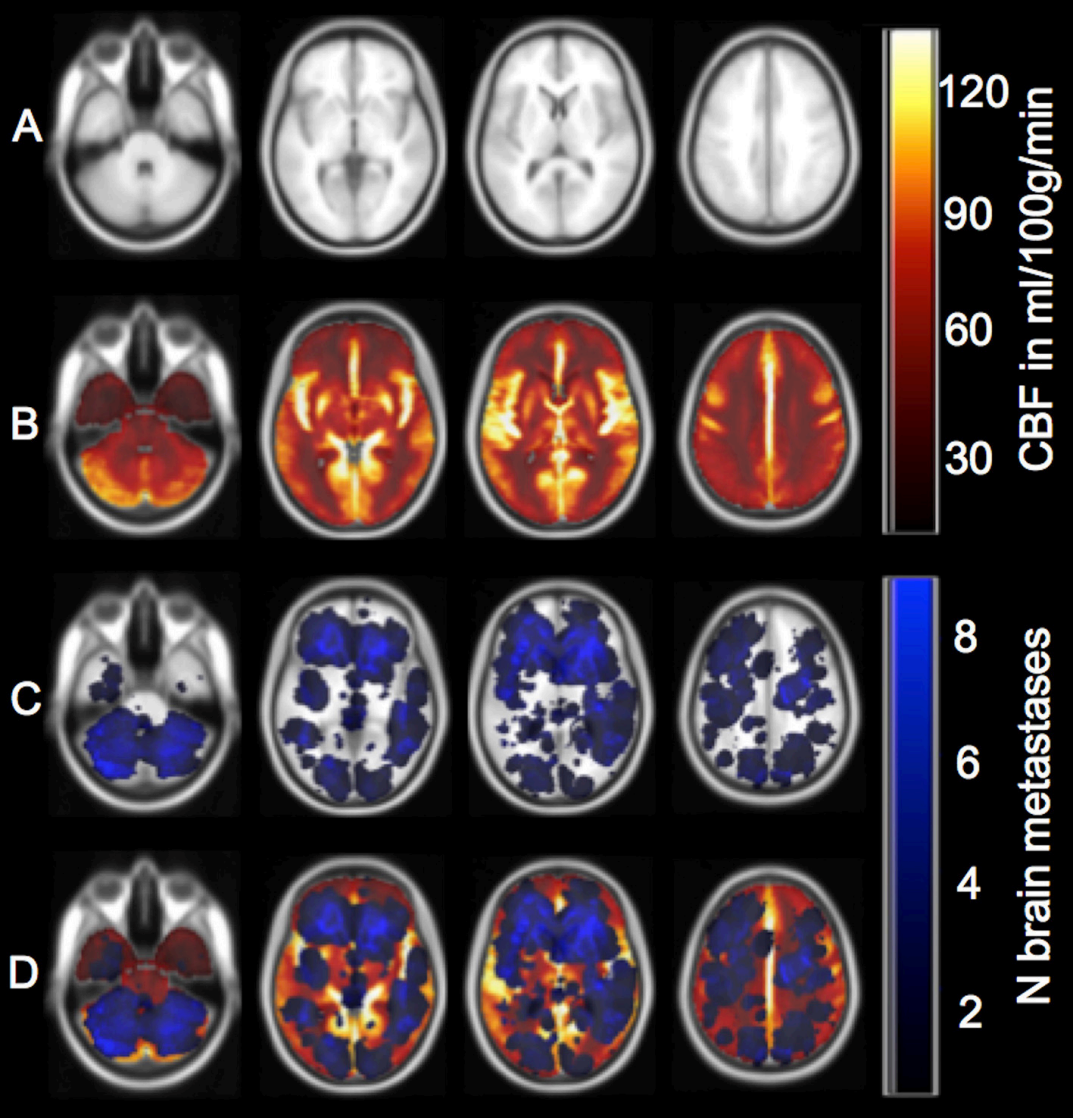
Overview of perfusion calculation from caudal to cranial (1–4). **(A)** Example of the 1 mm MNI brain that served as a template for co-registration of the patient's contrast-enhanced T1-weighted images and the CT perfusion map. **(B)** Display of the cerebral blood flow (CBF) map (see color scale on the upper right; light yellow also corresponds to CBF values ≥120 ml/100 g/min due to better visualization). Cerebral blood volume (CBV), mean transit time (MTT), and time to peak (TTP) maps are not depicted. **(C)** Cumulative brain metastases frequency map (see color scale on the lower right; light blue also corresponds to ≥8 brain metastases due to better visualization). **(D)** Overlay of the metastases sum map, the perfusion map, and the MNI brain. CBF, CBV, MTT, and TTP values were extracted for each voxel-group with and without a BM.

All perfusion raw data were processed in a central core-lab on a workstation dedicated for perfusion analysis with motion correction and low band temporal noise removal (Syngo mmwp VE52A with VPCT-Neuro; Siemens Healthcare, Erlangen, Germany). Non-parenchymal voxels corresponding to bone, vasculature, calcification, and cerebrospinal fluid were automatically excluded by adaptive intensity thresholding. Perfusion parameter maps were calculated based on a deconvolution model by least mean squares fitting (12). Perfusion data sets with erratic or incomplete arterial and venous attenuation time curves were excluded (*n* = 5). All calculated perfusion maps were reformatted to 1 mm Montréal Neurological Institute (MNI) standard space using the Oxford Centre for Functional Magnetic Resonance Imaging of the Brain (FMRIB) Software Library 5.0 (Analysis Group, Oxford, UK) with nearest neighbor interpolation so that quantitative image gray values remained unchanged (13). Mean voxel-wise perfusion parameter maps normalized to the MNI space were then used in our study cohort to obtain normal perfusion values for each voxel of the perfusion map and each voxel with a BM (Figures 1A–D).

### BM atlas

**Figure 2:**
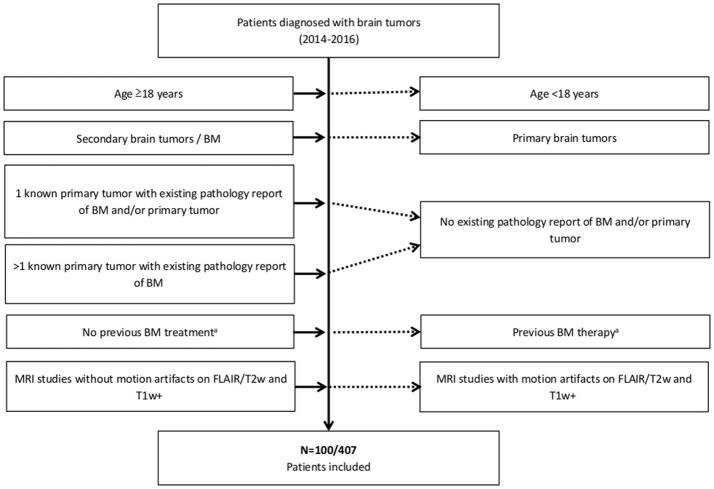
Summary of the inclusion **(Left)** and exclusion criteria **(Right)**. ^a^No surgery, radiotherapy, or chemotherapy (e.g., bevacizumab) for treatment of BM previous to MRI.

Since CT does not offer sufficient tissue contrast for reliable detection of BM we decided to delineate and define BM location by contrast-enhanced MRI due to its superior resolution (14). One hundred consecutive patients with different tumor entities screened between 01/2014 and 03/2015 in our university hospital fulfilled the inclusion and exclusion criteria given in Figure 2. In particular, all patients included were not known to have BM prior to MRI and were therefore untreated for BM.

### MRI protocol

The study was conducted using 1.5 Tesla MRI systems in 94 patients (Magnetom® Sonata, Siemens Healthcare, Erlangen, Germany; Magnetom® Symphony, Siemens Healthcare, Erlangen, Germany, or Magnetom® Avanto, Siemens Healthcare, Erlangen, Germany) and 3 Tesla MRI scanners in 6 patients (Magnetom+ Skyra, Siemens Healthcare, Erlangen, Germany or Ingenia, Philips Medical Systems, Best, The Netherlands).

The standard imaging protocol always included axial T1w spin echo (2D T1 SE+) with flow compensation and/or three-dimensional T1w gradient echo sequences (3D T1 GRE+) following weight-adjusted i.v. Gadolinium injection, respectively. If both sequences were acquired, axial 3D T1 GRE+ was processed for further analysis. Sequence parameters for the different MRI scanners are given in Table 1.

**Table 1:** Sequence parameters of the axial post-contrast T1w spin echo with flow compensation (2D T1 SE+) and/or three-dimensional T1w gradient echo sequence (3D T1 GRE+) of different MRI scanners.

**Variable/Sequence**	**Magnetom® Sonata**	**Magnetom® Symphony**	**Magnetom® Avanto**	**Magnetom® Skyra**	**Ingenia**
Tesla	1.5	1.5	1.5	3	3
No. of patients	11	25	58	2	4
**2D T1 SE**+					
TE (ms)	10	17	12	12	10
TR (ms)	460	690	556	550.0	600
Flip angle	70°	70°	70°	150°	70°
Matrix	192 × 256	192 × 256	192 × 256	432 × 512	432 × 432
FOV	172.5 × 230	172.5 × 230	172.5 × 230	194.1 × 230	230 × 230
Pixel size (mm)	0.9 × 0.9	0.9 × 0.9	0.9	0.4 × 0.4	0.5 × 0.5
No. of slices	20	25	25	32	28
Slice thickness (mm)	5	5	5	4	4
Interslice gap (mm)	6.5	6.0	6.5	4.4	5.0
**3D T1 GRE**+					
TE (ms)	4	2.44	5	2.5	4
TR (ms)	1900	5.33	9	1900	8
Flip angle	15°	10°	20°	9°	8°
Matrix	176 × 256	192 × 192	512 × 512	256 × 256	256 × 256
FOV	176 × 256	250 × 250	250 × 250	240 × 240	240 × 240
Pixel size (mm)	1 × 1	1.3 × 1.3	0.5 × 0.5	0.9 × 0.9	0.9 × 0.9
No. of slices	160	112	161	160	160
Slice thickness (mm)	1	1.4	1	1	1

*2D T1 SE+, 2-dimensional post-contrast T1-weighted spin echo with flow compensation; 3D T1 GRE+, 3-dimensional post-contrast T1-weighted gradient echo; FOV, field of view; TE, echo time; TR, repetition time*.

A necrotic BM was defined as central hypointense area surrounded by a rim of marked enhancement on post-contrast T1-weighted MR images.

### Segmentation of BM

In 100 patients we detected and semi-manually segmented a total of 809 BM. Semi-automated segmentation was performed using the Analyze Software System 11.0 (Biomedical Imaging Resource, Mayo Clinic, Rochester, MN, USA) (15). Afterwards, axial post-contrast T1-weighted MR images were automatically co-registered to the MNI 1 mm brain by using the FMRIB Software Library 5.0 linear (affine) registration tool. Correct registration of all post-contrast T1-weighted MR images and the segmented BM to the MNI brain was secured through visual inspection by two readers (T. S. and S. S.). From all BM masks in MNI space, a binary sum mask was created, subdividing the MNI brain in regions with (BM_pos_) and without BM (BM_neg_).

### Statistical analysis

Statistical analysis was conducted using IBM SPSS Statistics® software (IBM® 2011, version 20, Armonk, New York, USA) and R (The R Foundation, version 3.3.1 Vienna, Austria). The differences in CBF, CBV, MTT, and TTP between all BMpos and BMneg voxels were compared by the independent *t*-test since we had a large enough sample size. Voxels with more than one BM were weighted according to the number of BM occurring within the voxel. Mann-Whitney *U*-test was performed to determine voxel-wise differences of perfusion values among BM with and without central necrosis. If not otherwise indicated, data are given as median ± standard deviation.

## Results

### Descriptive statistics

Patient age at diagnosis of BM was 62 ± 12.5 years. The number of patients, number of BM, and number of BM with central necrosis for the whole group and for the different primary tumors are described in Table 2. Primary tumor entities were lung cancer (*n* = 51: 37 non-small lung cancer and 14 small cell lung cancer), skin cancer (*n* = 17: 16 melanoma and one Merkel-cell carcinoma), breast cancer (*n* = 10), genitourinary cancer (*n* = 10: one choriocarcinoma, two prostate, two kidney, three urothelial cell cancer, and two testicular cancer), cancer of unknown primary (*n* = 6), gastrointestinal cancer (*n* = 4: two colon and two rectal cancer), and sarcoma (*n* = 2).

**Table 2:** BM characteristics among the whole cohort and the different primaries.

**Variable**	**All**	**Lung**	**Skin**	**Breast**	**GU**	**CUP**	**GI**	**Sarcoma**
No. of patients	100	51	17	10	10	6	4	2
No. of BM	809	312	199	146	42	47	41	22
• Supratentorial	580	191	187	71	32	43	35	21
• Infratentorial	229	121	12	75	10	4	6	1
Necrotic changes of BM	184	88	29	11	16	21	6	13

*BM, brain metastasis; CUP, cancer of unknown primary; GI, gastrointestinal primary tumor; GU, genitourinary primary tumor; No., number*.

### Perfusion patterns

Among all patients, CBF (56 ± 34 vs. 59 ± 35 ml/100 g/min, *p* < 0.001) and CBV (3.7 ± 2.0 vs. 3.9 ± 2.1 ml/100 g, *p* < 0.001) were lower and MTT (4.8 ± 0.7 vs. 4.7 ± 0.7 s, *p* < 0.01) and TTP (9.3 ± 0.9 vs. 9.2 ± 0.9 s, *p* < 0.001) were higher in BM_pos_ than within BM_neg_.

**Figure 3:**
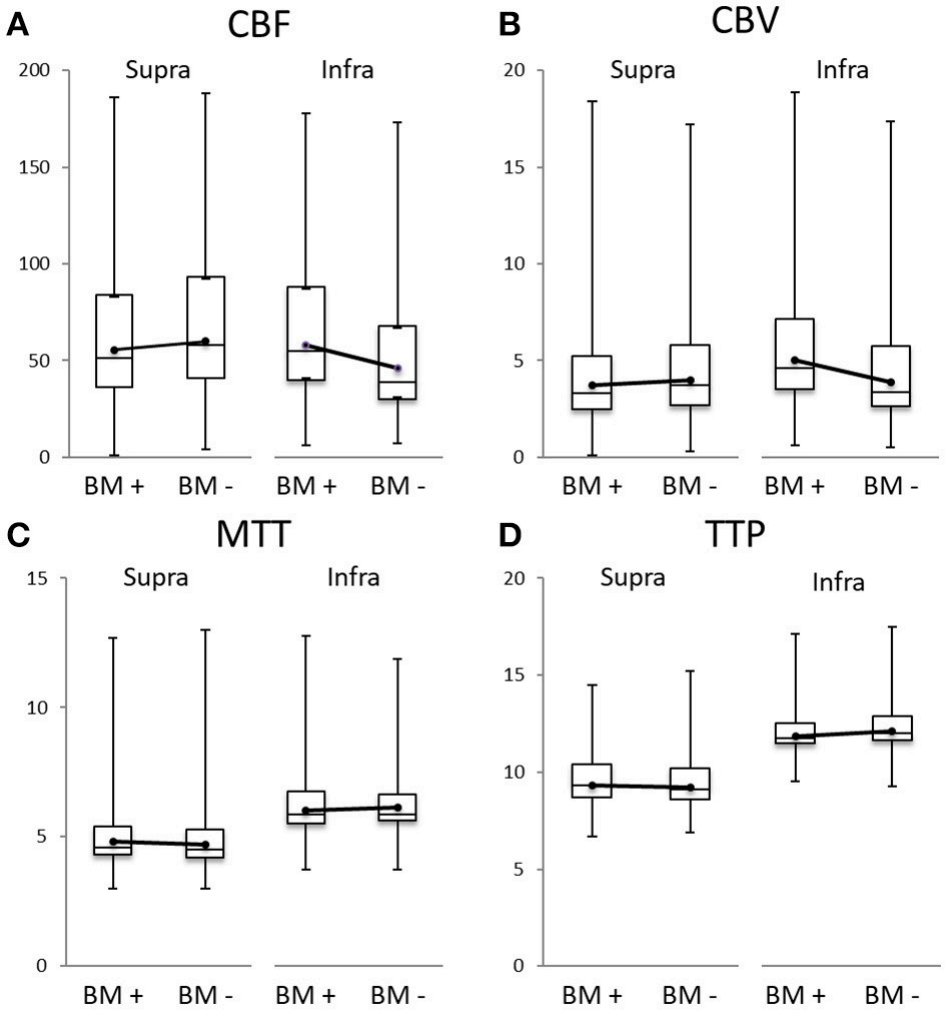
Boxplots of cerebral blood flow (CBF in ml/100 g/min, **A**), cerebral blood volume (CBV in ml/100 g, **B**), mean transit time (MTT in s, **C**), and time to peak (TTP in s, **D**) of the supra- (left side of each panel) and infratentorial area (right side), always for the brain metastases (BM_pos_ = BM +) and their corresponding area without any BM (BM_neg_ = BM –). The dots represent the mean. For better comparability between BM_pos_ and BM_neg_, they are connected by a line.

When studying perfusion values for the supra- and infratentorial area separately, we found a marked difference: supratentorial BM occurred more often in areas with lower CBF (51 ± 33 vs. 58 ± 35 ml/100 g/min, *p* < 0.001) and CBV (3.3 ± 1.9 vs. 3.7 ± 2.1 ml/100 g, *p* < 0.001) and higher MTT (4.6 ± 0.8 vs. 4.5 ± 0.8 s, *p* < 0.001) and TTP (9.3 ± 1.1 vs. 9.1 ± 1.1 s, *p* < 0.001) indicating lower perfusion compared to BM_neg_. On the contrary, infratentorial BM favored areas with higher CBF (55 ± 33 vs. 39 ± 29 ml/100 g/min, *p* < 0.001) and CBV (3.7 ± 2.0 vs. 2.7 ± 1.9 ml/100 g, *p* < 0.001) and shorter MTT (4.7 ± 0.7 vs. 4.7 ± 0.6 s, *p* < 0.001) and TTP (9.4 ± 0.6 vs. 9.6 ± 0.7 s, *p* < 0.001) implying higher perfusion compared to BM_neg_ (Figures 3A–D).

Supratentorially, these differences were found to be greater when adjusting for the number of BM occurring within a voxel: in voxels containing a high number of BM the median CBF was even lower compared to the median CBF in all supratentorial voxels.

**Figure 4:**
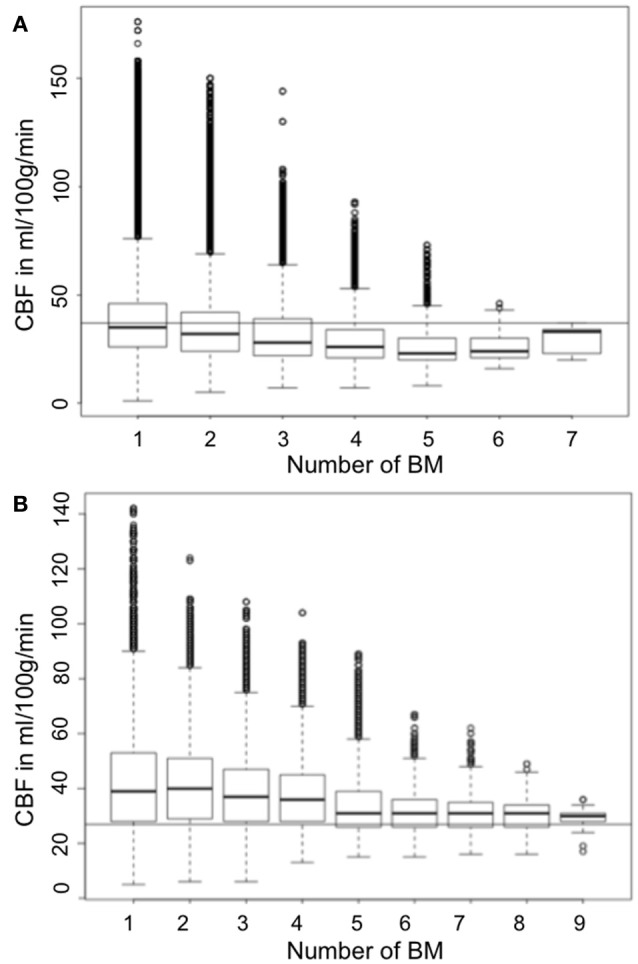
Boxplot showing the median CBF (y-axis) according to number of BM (x-axis) in relation to the median CBF **(A)** supra- and **(B)** infratentorial (continuous horizontal line).

On the contrary, the highest CBF values infratentorially were observed in voxels containing less BM (Figures 4A,B).

### Perfusion and central necrosis

BM with central necrosis showed a lower CBF (53 ± 33 vs. 59 ± 35 ml/100 g/min, *p* < 0.001) and CBV (3.5 ± 2.0 vs. 3.9 ± 2.1 ml/100 g, *p* < 0.001) and higher MTT (4.8 ± 0.8 vs. 4.7 ± 0.7 s, *p* = 0.002) compared to BM without necrosis. The difference for TTP was not significant (9.4 ± 0.6 vs. 9.3 ± 0.6 s, *p* = 0.182).

## Discussion

In a cohort of 100 untreated BM patients we were able to demonstrate an inverse relationship between BM location and brain perfusion depending on lesion location. BM development in proportionally higher perfused areas occurred infratentorially whereas supratentorial BM favored lower-perfused areas. Supratentorial, these results were strengthened by our finding that the difference in perfusion increased with the number of metastases per brain voxel. Furthermore, we found that BM with central necrosis more often developed in areas with lower blood flow that corresponds to the pathophysiology of tumor necrosis.

The observed differences in BM location were greater for CBF and CBV and only marginal for MTT and TTP. This is likely due to the greater physiological heterogeneity of CBF and CBV between anatomical and structural brain regions whereas MTT and TTP values of the gray and white matter are comparatively similar and do not show large variance throughout the brain (16). But what structural differences may explain our opposite supra- and infratentorial perfusion patterns? One important difference is that in evolutionary terms, the cerebellum and brainstem are older compared to the cerebral neocortex what involves a characteristic vascular architecture (17). De Reuck et al. showed that there is only one type of cerebellar cortical arterial branches whereas the vascularization of the neocortex is more complex and the type of cortical branch is cell layer-dependent (18). Furthermore, one could hypothesize that the perfusion differences are primary due to hemodynamic mechanisms. The vessel cross sectional area of the vertebral arteries is much smaller compared to the internal carotid arteries; therefore, less blood volume arrives infratentorially. Hence, higher perfusion might be a requirement for the development of cerebellar BM. We found the differences in median CBF and CBV of BM_pos_ and BM_neg_ for infratentorial BM being much greater compared to supratentorial BM what may support this hypothesis further.

Preclinical studies showed that the process of metastastic spread to the brain in lung adenocarcinoma and breast cancer cells requires production of vascular endothelial growth factor-A (VEGF-A) that induces early neoangiogenesis whereas metastatic melanoma cells were shown to proliferate along pre-existing and remodeled vessels (vessel cooption) independently from VEGF-A (19–21). Hence, our study findings could indicate that supratentorial BM rely more on VEGF-A-mediated mechanisms of angiogenesis compared to infratentorial BM.

## Limitations

The main limitation of our study—since the results imply a relationship between brain perfusion and the occurrence of BM—is that it is unclear whether the perfusion preferences also existed in the individual BM patient because it was derived from a mean perfusion map template. For this purpose, additional perfusion imaging on serial brain MRI for staging purposes could be performed in future clinical studies. However, since Nudelman et al. already demonstrated alterations of the cerebral perfusion in non-metastatic breast cancer patients, this will be difficult to interpret (9). In addition, interindividual as well as longitudinal comparison would be limited.

Our measured perfusion parameter differences are partially very subtle (yet significant) rather for MTT/TTP than for CBF/CBV and thus raise the question about the clinical relevance for an individual BM patient when observing the entire cohort. The following separation into supra- and infratentorial BM showed that the inverse behavior of infratentorial BM amortized initial larger perfusion value differences. Thus, we believe that our findings do have clinical significance, especially when looking for a supra- or infratentorial BM distribution. Interestingly, our median infratentorial CBV was 3.7 ml/100 g in BM_pos_ and 2.7 ml/100 g in BM_neg_ whereas the ischemia CBV threshold was reported to be 2.0 ml/100 g (16, 22, 23).

As we considered this a proof of concept study, we refrained from analyzing differences in primary tumor entities and histological or molecular subtypes. Since, for example, epidermal growth factor receptor in lung cancer was shown to influence the preferred anatomical distribution of BM, future studies should elucidate the influence of those factors on the perfusion pattern or vice versa (24).

## Conclusions

We demonstrated that supratentorial BM developed preferentially in areas with low-normal perfusion, compared to BM of the cerebellum and brainstem, which primarily established growth in areas of high perfusion. Our findings point to diverse growth characteristics of supra- and infratentorial BM, which could be based on a differential vascular supply, hemodynamic requirements, production of pro-angiogenic factors, and/or anatomical homing behavior of different types of BM. Further characterization of those processes could lead to earlier detection of BM and may even provide a novel therapeutic approach.

## Author contributions

Conceptualization: TS, AK, GS, and SG. Investigation: TS, AK, JS, KP, MG, GS, MM, JF, and SG. Methodology: TS, AK, GS, and SG. Software: AK and SG. Writing ± original draft: TS, AK, JS, and SG. Writing ± review & editing: TS, AK, JS, KP, MG, GS, MM, JF, and SG.

### Conflict of interest statement

The authors declare that the research was conducted in the absence of any commercial or financial relationships that could be construed as a potential conflict of interest.
